# Sickle-shaped high gain and low profile based four port MIMO antenna for 5G and aeronautical mobile communication

**DOI:** 10.1038/s41598-023-42457-8

**Published:** 2023-09-21

**Authors:** Ammar Armghan, Sunil Lavadiya, Pamula Udayaraju, Meshari Alsharari, Khaled Aliqab, Shobhit K. Patel

**Affiliations:** 1 Department of Electrical Engineering, College of Engineering, Jouf University, 72388 Sakaka, Saudi Arabia; 2 Department of Information Communication and Technology, Marwadi University, Rajkot, 360003 Gujarat India; 3 Department of Computer Science and Engineering, SRKR Engineering College, Bhimavaram, 534204 Andhra Pradesh India; 4 Department of Computer Engineering, Marwadi University, Rajkot, 360003 Gujarat India

**Keywords:** Electrical and electronic engineering, Applied physics, Electronics, photonics and device physics

## Abstract

The construction of the four-port MIMO antenna in the form of a sickle is provided in the article. Initially, the single port element is designed and optimized. Next, a structure with two ports is created, and lastly, a design with four ports is completed. This process is repeated until the design is optimized. Three types of parametric analysis are considered, including variations in length, widths of sickle-shaped patches, and varying sizes of DGS. The frequency range of 2–8 GHz is used for structural investigation. The − 18.77 dB of return loss was observed at 3.825 GHz for a single-element structure. The optimized one-port structure provides a return loss of − 19.79 dB at 3.825 GHz. The port design offers a bandwidth of 0.71 GHz (3.515–4.225). The four-port design represents two bands that are observed at 3 GHz and 5.43 GHz. Both bands provide the return loss at respectively − 19.79 dB and − 20.53 dB with bandwidths of 1.375 GHz (2.14–3.515) and 0.25 GHz (5.335–5.585). The healthy isolation among both transmittance and reflectance response is achieved. The low-profile material was used to create the design that was presented. The article includes a comparison of the findings that were measured and those that were simulated. The four-port design that has been shown offers a total gain of 15.93 dB, a peak co-polar value of 5.46 dB, a minimum return loss of − 20.53 dB, a peak field distribution of 46.43 A/m and a maximum bandwidth of 1.375 GHz. The values for all diversity parameters like ECC are near zero, the Negative value of TARC, Near to zero MEG, DG is almost 10 dB, and a zero value of CCL is achieved. All diversity parameter performance is within the allowable range. The design is well suited for 5G and aeronautical mobile communication applications.

## Introduction

The fast growth of wireless devices, limited bandwidth, and restricted channel capacity have considerably increased the number of people trying to establish improved communication network standards in the current day. As a direct consequence of this, the development of communication systems of the next generation, known as 5G, using the mm-wave spectrum and boasting much-increased channel capacity and data speeds, has been sped up^1^. By addressing the growing need for more reliable connections, more effective data rates, and reduced power consumption from the Internet of Things (5G), the future of innovative technologies like virtual reality and intelligent cities becomes brighter^2^. Using a single antenna in the mm-wave spectrum presents substantial difficulties due to signal fading, atmospheric absorptions, and route loss attenuations^3^. The Multiple-Input Multiple-Output (MIMO) antenna has been highlighted as a key enabling technology for current and future wireless systems due to its ability to use multiple antennas to increase channel capacity and enable high data rates and throughput on the scale of gigabits per second^4^.

Fifth-generation MIMO antennas are more compact, which aids in assimilation in MIMO systems. Concurrent operation is made possible by the wide bandwidth, while the high gain reduces atmospheric attenuations and absorptions at mm-wave frequencies. Making components with minimal mutual coupling and high isolation, which improves antenna performance, is also challenging in MIMO antenna design^5^. The following are recent developments in antenna technology for 5G networks operating in mm-wave frequencies. The significant atmospheric and propagation losses at mm-wave frequency are beyond antenna designs' capabilities at those bands with a modest gain. Several high gains and beam-steering antenna array systems with high signal strength and comprehensive coverage have been proposed to counteract these attenuation^6,7^. In reference^8^, it is stated that a PIFA array in MIMO configuration may achieve a peak simulated gain of 12dBi across a 1 GHz operating bandwidth. Reference describes a MIMO antenna built on an EBG bandwidth-wise (0.80 GHz). When supplied from a single port, however, multi-element antenna arrays match the performance of a single antenna. However, MIMO antennas show multipath propagation with 5G's hallmarks of enhanced data throughput, capacity, and connection dependability. Many MIMO antenna designs for 5G mm-wave applications have recently been published^9^. The maximum gain of 7.41dBi is attained at 28 GHz with a bandwidth of 1.5 GHz between 27.2 and 28.7 GHz, thanks to the antenna’s design. An article on the DRA-based concept for 5G communication is reported in^10^, although they only support a maximum data rate of around 1 GHz. Reference^11^ proposes a multi-element antenna design optimal for 5G communications due to its ability to direct radiation in the desired oblique direction. ECC is also tested to guarantee MIMO performance^12^. The SIW feeding method has a maximum gain of 7.37, as described in^10^. The suggested antenna in^13^ operates between 24.25 and 28.35 GHz, covering both 5G bands, with a gain that ranges from 8.2 to 9.6 dBi. In addition, a 12 × 50 × 80.8 mm^3^ four-element T-shaped MIMO antenna is demonstrated for 5G applications. Similarly, reference^14^ reports on a slotted MIMO antenna array fed by an SIW for mm-wave communication. The most significant gain achieved by the suggested MIMO architecture is 8.732 dB, and its resonant frequency is 25.2 GHz. The effects of diversity gain, MEG, and ECC are also investigated. For 5G networks, a similar 2-Port MIMO array has been disclosed^15^.

The notion entails the development of a specialized planar structure with chiral components that exert control over the phase of circularly polarized microwaves. The aforementioned surface retains the distinct circular polarization state of microwaves, allowing the generation of distinct and precise holographic representations for various objects or scenes when exposed to microwaves, all falling within the microwave frequency spectrum. The aforementioned breakthrough exhibits possibilities for utilization in sophisticated imaging systems, communication technologies, and microwave-based technologies that need meticulous regulation of polarization and imaging capabilities^16^. Dispersion is essential to optical system performance. Recent advances have made the artificial Meta surface a promising dispersion modification alternative. The current meta-atom dispersion manipulation idea only regulates the propagation phase within the operating frequency range or a few wavelengths. This work introduces the chirality-assisted phase to manipulate meta-atom dispersion. This method’s theoretical demonstration is also given. A detailed investigation of the chiral meta-atoms reflective mode dispersion features is given, focusing on its frequency range. This paper proposes and builds two hybrid dispersion-engineered metal mirrors (HDEMs) to demonstrate dispersion manipulation at various frequencies. HDEMs are intended for achromatic lower-half band focusing and highly dispersive upper-half band focusing. HDEMs also have excessive dispersive focusing in the bottom half band and aberrant focusing in the top half band. Full-wave simulation and measured results prove the technique’s validity and applicability. This work uses a new degree of freedom to alter dispersion to construct Meta surfaces with customized dispersion^17^. The suggested design in^18^ has a succession of fractally loaded circular patch antenna components on top and a partially grounded, protruding T-shaped stub on the substrate's bottom. Using four triangular slots on the substrate and a T-shaped stub on the ground, sufficient isolation over the appropriate bands is achieved. The recommended design has a succession of fractally loaded circular patch antenna components on top and a partially grounded, protruding T-shaped stub on the substrate’s bottom. Using four triangular slots on the substrate and a T-shaped stub on the ground, sufficient isolation over the appropriate bands is achieved.

Article^19^ suggests no extra decoupling mechanism is required for the design to ensure isolation between many ports. By merging four single wideband antenna components, a metallic disc with a circular shape is formed in the ground plane of the proposed antenna. With a 180° phase difference, this disc acts as a current pool that isolates individual ports. The lotus-shaped array-based configuration using low-profile material can be used for 5G communication applications. The MIMO antenna comprises a lotus-shaped radiating element, an in-ground DGS and extra slits to facilitate boresight radiation. In both the 4 × 4 and 8 × 8 designs, all radiating elements are organized in an orthogonal manner. To achieve a high isolation level exceeding 16 dB, a distinctive ground-based decoupling structure is employed^20^. The advancement involves creating a MIMO antenna with an arc-shaped design featuring two ports, offering improved isolation characteristics. To achieve wideband performance for this antenna, a smaller ground plane is employed^21^.

To enhance the isolation between the MIMO antenna elements while keeping complexity and cost low, the antenna elements are aligned in an orthogonal configuration with a spacing of 0.3λ_o_ between them. This setup incorporates various components such as EBG structure, capacitive elements (CE), DGS, and neutralization line (NL). Additionally, an "EL" slot is integrated into the radiating element, and two identical stubs are connected to the partial ground to enhance impedance matching and radiation characteristics across the desired frequency bands^22^. The SRR-based symmetrical partial ground approach provides a peak gain of 10.6 dBi. The suggested antenna design can span an extensive frequency range (25.1–37.5 GHz). An 8 × 8 MIMO antenna for 5G devices is shown in another article^1^, having a substrate size of 31.2 × 31.2 × 1.57 mm^3^. Similarly^23^ represents a 5G MIMO antenna with interconnected metamaterial arrays. The reported EBG-reflector-based antenna system boasts a high gain of up to 11.5 dB and a broad operational frequency range. The suggested MIMO setup's ECC and diversity gain analysis are observed. Article^24^ describes a high-gain antenna based on the Fabry–Perot interferometer. The proposed design can achieve a peak gain of 14.1 dBi at mm-wave frequencies between 26 and 29.5 GHz. Error correction code (ECC) analysis also assesses how well the proposed antenna arrangement performs in a MIMO setting. In reference^9^, a two-element MIMO DRA is presented. It presents 5G networks. The described antenna operates in the frequency range of 27.19–28.48 GHz. The 27.19–28.48 GHz band is optimal for 5G because of its high gain. The shown antenna achieves a gain of about 10 dB.

The Internet of Things (IoT), using industrial, scientific, and medical (ISM) bands like 2.45 and 5.8 GHz, facilitates remote sharing of heart data from pacemakers to medical professionals. This research introduces the first-ever communication between a compact dual-band MIMO antenna inside a leadless pacemaker and an external dual-band MIMO antenna on the body within ISM frequency bands^25^. Article^26^ introduces a straightforward yet effective eight-element MIMO antenna array that achieves broadband coverage from 3.3 to 6 GHz, tailored for fifth-generation (5G) smartphones. The antenna's simplicity is attributed to its multiple-mode structure, incorporating double coupled-fed loop modes and a slot mode activated through the feeding strip linked to the tuning branch. Ingeniously, the inclusion of the tuning branch on the side frames obviates the need for additional lumped elements, streamlining the antenna’s design. Moreover, the implementation of polarization diversity techniques is harnessed to enhance isolation performance. Article^27^ suggested configuration comprises a singular module, featuring an adapted L-shaped rectangular radiator combined with a Z-shaped slot-loaded defected ground structure (DGS). Optimization of the DGS employs machine learning algorithms to enhance the achievable maximum absolute resonant bandwidth (ARBW) output. This is accomplished by fine-tuning the defected ground structure using Right Shifting (RS) and Left Shifting (LS) techniques, while simultaneously acquiring input features.

Compact and low-profile dual-band 2 × 2 wideband wearable slot antenna designed for the 3.4–3.6 GHz frequency range (LTE Band 42) to be integrated into a smartwatch. The antenna is optimized to meet the size constraints of smartwatch applications while also ensuring resonance with the upcoming 5G technology represented in Article^28^. Article^29^ is a dual-band multiple-input multiple-output (MIMO) antenna designed using metamaterials to achieve high isolation, intended for millimeter-wave communication networks in the context of 5G. The antenna takes the form of a pentagon-shaped monopole, delivering a dual-band performance across the 5G 28/28 bands and boasting a wide operational bandwidth. The four-port MIMO antenna is crafted for 5G-NR band applications, encompassing n77, n78, and n79 frequencies. The MIMO antenna's development trajectory is explored by drawing insights from the characteristics and optimized single-element antenna. Measurement results show a remarkably high impedance match for the MIMO setup and increased isolation within the same band, enhancing the 5G-NR bandwidth performance^30^.

The key novelty points of the proposed design structure are as follows.

- The suggested design is a high gain that employs low-profile material with strong MIMO properties.
- The small size and square shape, the proposed MIMO antenna is easily integrated into 5G devices.
- The disclosed antenna's strong MIMO performance vouchsafes the design’s viability for 5G wireless communications in the future.
- The design cost of the presented structure is very low due to the FR4-based dielectric material.
- Sufficient isolation by using passive strips between the radiating elements.
- Performance of different diversity parameters like ECC, TARC, MEG, DG and CCL is satisfactory.
- Comparison among simulated and measured results authenticates the response of the design.
- Design is compared with other existing designs and it represents design performance is good.

The introduction is represented in section “Introduction”, The design and modelling of the planned design structure are presented in section “Four port antenna design and modelling”, the Outline of the intended design structure is described in section “Performance observation of the proposed design”, and the concluding remarks are represented in section “Conclusion”. The performance index regarding return loss, gain, bandwidth, and directivity is incorporated into the manuscript, and the structure highlight is compared with other articles. The low-profile 4-element MIMO antenna array can use mm-wave 5G frequency ranges for 5G devices such as mobile WiFi and smartwatches. The resonating response at 3 GHz enables marine radio navigation and amateur radio and 5.43 GHz will be used for earth exploration satellites, space research and aeronautical radio location applications.

## Four port antenna design and modelling

**Figure 1:**
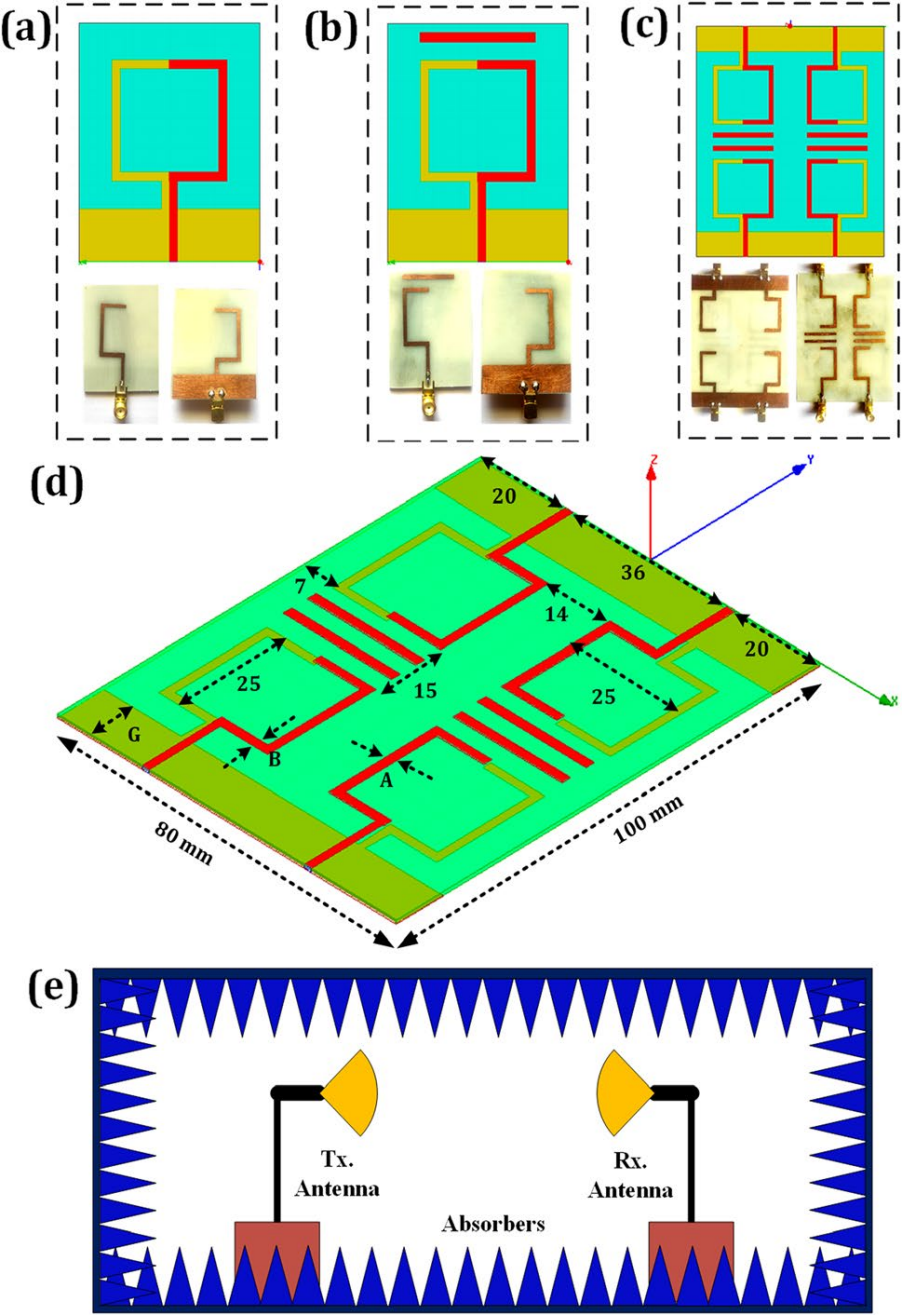
(**a**) Proposed single patch element structure. (**b**) Optimized single-element design. (**c**) Proposed four-element MIMO antenna. (**d**) Three-dimensional view of presented design. (**e**) Measurement setup of the proposed MIMO antenna structure.

Figure 1 depicts the suggested antenna structure design. The dimension of the invention is 100 × 80 × 1.6 mm^3^. The finite element method is used to model the proposed antenna structure. There are two stages involved in finalizing the single-element design. The planned single-element design is presented in Fig. 1a. The optimized design of one element-based MIMO antenna is shown in Fig. 1b. The four port-based MIMO design is represented in Fig. 1c.

After doing multiple iterations, the shape of a sickle-shaped patch is considered. A parasitic patch in a MIMO antenna system serves numerous roles and offers various benefits. It plays a role in beam steering by altering the radiation pattern and improving signal coverage and reception in specific directions. It also helps reduce mutual coupling between antenna elements, enhancing isolation and minimizing interference. The addition of a parasitic patch can increase antenna gain, improve directivity, and enhance overall MIMO performance by maintaining channel diversity.

Additionally, the compact design of parasitic patches makes them suitable for integration into small form factor MIMO antennas, making them valuable in space-constrained applications^31^. The parasitic patch elements are loaded between two patch-radiating parts. The thickness of the parasitic patch is 2 mm. The length of the parasitic patch is 25 mm. The thickness of all radiating patch structures is 2 mm. The spacing among the two patch elements is 36 mm. The feed line's spacing from the structure’s outer is 20 mm. The patch, parasitic element and ground layers are considered copper material. The FR4 epoxy is chosen as a substrate. The FR4 provides a dielectric constant of 4.4^32^. The proposed design is tested in an anechoic chamber, as shown in Fig. 1e. The prototype design has a patch structure, ground plane and feedline. Using Eqs. (1)–(3), we can determine the patch antenna's width and length at its resonant frequency^33^.

1 $$ W = \frac{c}{2f}\left( {\frac{{\varepsilon_{r} + 1}}{2}} \right)^{{ - \frac{1}{2}}} $$

Here, relative permittivity is represented by, Resonating frequency is represented by $f$, and speed of light is represented by $c$. The impedance matching is achieved by proper width selection. The consideration of 0.5$\lambda_{e}$ is taken for the patch length selection. The $\lambda_{e}$ can be calculated using Eq. (2). The edge sorting will lead to a variation in the length of structure^34,35^.

2 $$ \lambda_{e} = \frac{c}{{f\sqrt {\varepsilon_{e} } }} $$

3 $$ L = \lambda_{e} - 2\Delta L $$

4 $$ \varepsilon_{e} = \frac{{\varepsilon_{r} + 1}}{2} + \frac{{\varepsilon_{r} - 1}}{2}\left( {1 + 12\frac{h}{W}} \right)^{{ - \frac{1}{2}}} $$

5 $$ .412h\frac{{\left( {\varepsilon_{e} + 0.3} \right)\left( {\frac{W}{h} + 0.264} \right)}}{{\left( {\varepsilon_{e} - 0.258} \right)\left( {\frac{W}{h} + 0.8} \right)}} $$

where the Wavelength of structure in guided media is represented by the effective dielectric constant. Equation (4) is used to calculate $\varepsilon_{e}$. The fringing effect will vary the effective length of the structure, and it can be calculated using Eq. (5).

## Performance observation of the proposed design

Analysis and design of high-frequency-operated antennas rely heavily on S-parameters. Antennas are essential components in wireless communication systems and are subject to complex electromagnetic interactions at high frequencies. S-parameters provide valuable insights into the behaviour of antennas, including their impedance matching, radiation patterns, and overall performance. By measuring or simulating S-parameters user can assess the antenna's impedance characteristics, reflection and transmission of signals. S-parameters play a vital role in the analysis, and design of high-frequency operated antennas, enabling engineers to achieve superior performance and reliability in wireless communication systems^36^. The impedance bandwidth is the parameter that needs to be taken into consideration for bandwidth calculation. The lower and upper frequencies that adhere to the necessary VSWR are determined by the frequency band on which the antenna operates. The frequency range across which the VSWR is less than 2 is referred to as the operating bandwidth. In the presented work VSWR is chosen for the criteria of 2:1.

Parametric iterations in MIMO antenna design provide the benefits of optimizing antenna performance, customization for specific applications, efficient resource utilization, comprehensive trade-off analysis, and enhanced structure robustness. Through iterative adjustments of antenna parameters, researchers can fine-tune the design to meet desired performance metrics, adapt to varying conditions, maximize resource utilization, analyze trade-offs, and ensure reliable operation in challenging environments. There are three types of parametric iterations are considered to achieve the optimum performance of the design.

**Figure 2:**
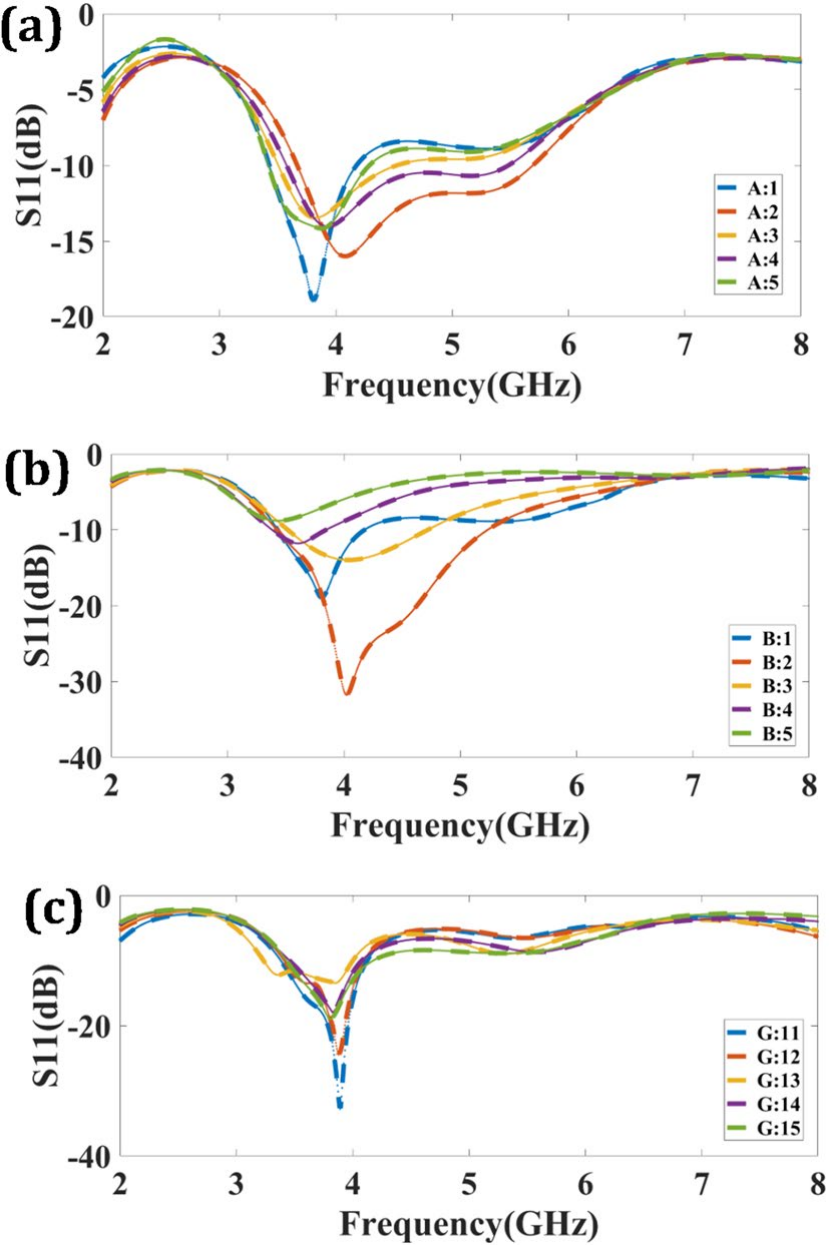
Parametric response of the planned design. (**a**) Patch length variation over 1–5 mm. (**b**) Patch thickness variation over 1–5 mm. (**c**) Ground region length variation over 11–15 mm.

The first parametric parameter is to vary the patch width (A). The variation of A is considered over the 1–5 mm is observed in Fig. 2a. The S_11_ is affected due to the thickness variation of the patch region. The return loss of − 17 dB is achieved for considering 2 mm thickness, and the maximum broadband response is observed for the 5 mm size of 2.135 (3.775–5.91). To make a uniform design structure the 2 mm size is taken into consideration. The second parametric consideration is by changing the length of the patch (B) region over the 1–5 mm is observed in Fig. 2b. The optimum response regarding S_11_ and sufficient bandwidth is kept for the 2 mm size. The 2 mm size represents a return loss of − 31.86 dB and a bandwidth of 1.81 GHz (3.425–5.235). The third parametric iteration in terms of varying lengths of the ground region (11–15 mm) is observed in Fig. 2c. The optimum return loss for an 11 mm size of the ground region is attained. The optimum result represents − 34 dB return loss at 4 GHz. Based upon consideration of optimum performance in terms of S_11_ and bandwidth value of A, B and G, chosen as 2 mm, 2 mm and 11 mm.

The validation of antenna performance through simulation and measurement is crucial for multiple reasons. Simulations enable engineers to theoretically analyze antenna behavior, facilitating the design, optimization, and troubleshooting of antenna systems before physical implementation. Simulations offer valuable insights into antenna performance across different scenarios by evaluating parameters like radiation patterns, impedance matching, and gain. However, simulations are based on assumptions and simplifications that may not accurately capture real-world complexities. Hence, it becomes essential to measure the actual performance of antennas to validate the simulated results and ensure their practicality. Measured data allows engineers to verify the antenna's real radiation characteristics, validate the affected models, account for environmental factors, identify any deviations, and fine-tune the design for optimal performance in real-world settings. Ultimately, employing a combination of simulation and measurement provides a comprehensive understanding of antenna behavior, thereby enhancing the reliability and effectiveness of antenna systems^37,38^.

**Figure 3:**
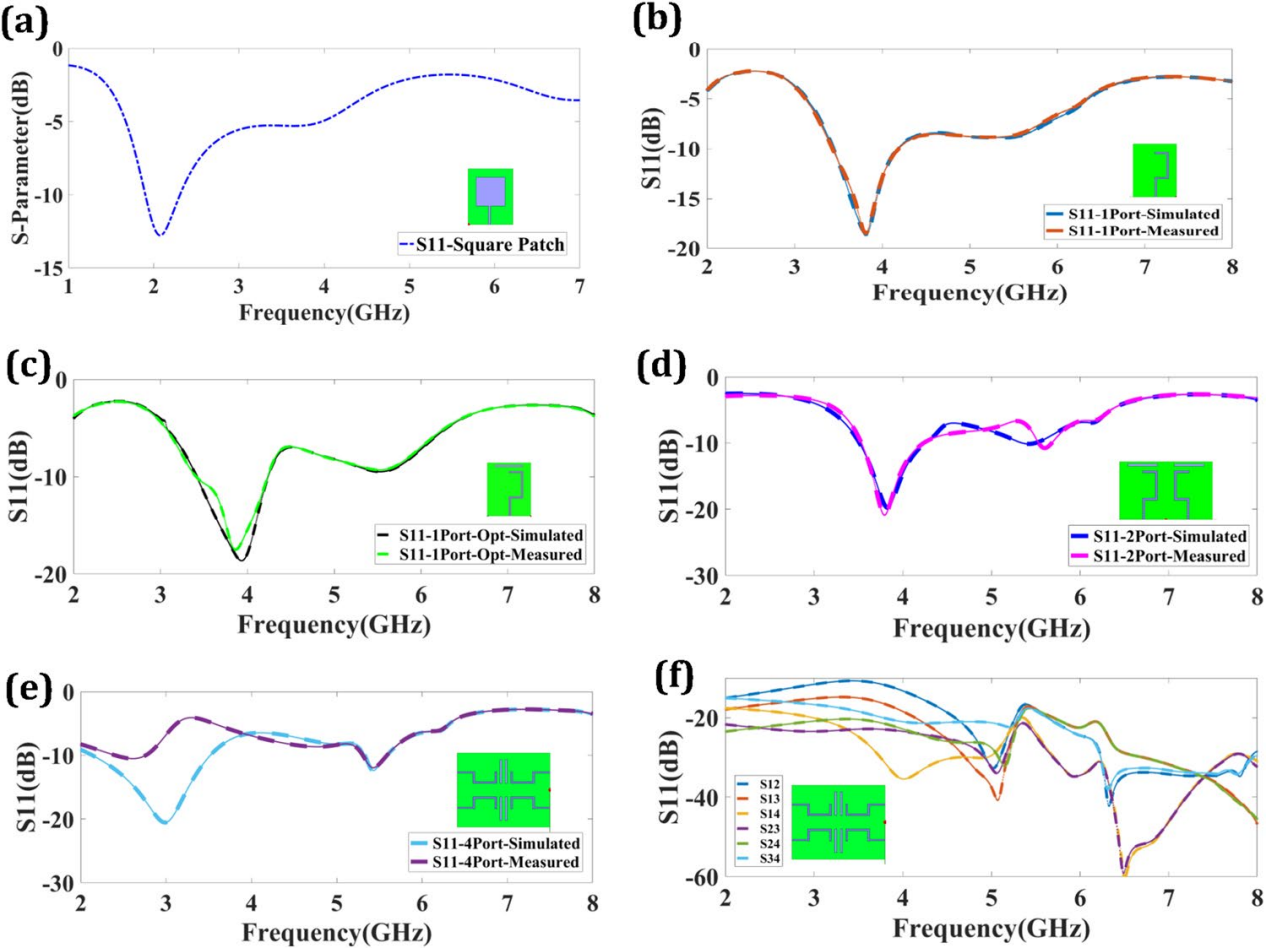
Return different proposed design structures. (**a**) Simple square patch-shaped design. (**b**) One port design. (**c**) Optimized one port design. (**d**) Two port MIMO antenna design. (**e**) Four port MIMO antenna design. (**f**) Scattering parameters for observing isolation.

Figure 3 represents a return loss response of different structures. Figure 3a represents the return loss of a simple square-shaped patch structure. The structure provides a − 13 dB response at 2.01 GHz. The return loss is enhanced in the proposed design compared to the simple design. The return loss of one port structure is shown in Fig. 3b. The − 18.77 dB of return loss was observed at 3.825 GHz. The one-port design structure represents a bandwidth of 0.7 GHz (3.4–4.1). The return loss of the optimized one-port structure is represented in Fig. 3c. The two-port structure provides a minimum return loss of − 19.79 dB at 3.825 GHz, illustrated in Fig. 3d. The port design offers a bandwidth of 0.71 GHz (3.515–4.225). As the number of ports enhances, the multiband response is observed for the four-port design structures. Four-port design is represented in Fig. 3e. The two bands are observed at 3 GHz and 5.43 GHz. Both bands provide the return loss at respectively − 19.79 dB and − 20.53 dB with bandwidths of 1.375 GHz (2.14–3.515) and 0.25 GHz (5.335–5.585). Figure 3f shows the comparison of scattering parameters. The healthy gap between the transmittance response and reflectance response indicates good isolation among both of them. The more than 20 dB of isolation near the peak is observed. The comparison of simulated and measured results authenticates the possibility of design.

There may be differences between the simulated and measured findings while simulating and measuring antennas owing to numerous variables that induce tolerance. These issues include manufacturing tolerances, material qualities, ambient circumstances, and measuring instrument constraints. Tolerances in manufacturing, such as differences in dimensions, material attributes, and fabrication procedures, may cause departures from the original design. Material parameters like conductivity and permittivity may also deviate from ideal values. Furthermore, environmental factors such as electromagnetic interference and the presence of surrounding objects might impact the antenna’s effectiveness. Finally, measuring equipment limitations, such as intrinsic noise, calibration errors, and bandwidth constraints, brings uncertainty into the estimated data. These variables contribute to the observed tolerance between simulated and measured antenna performance^39,40^.

The gain response of antennas plays a crucial role in MIMO systems that utilize multiple antennas. MIMO technology uses multiple antennas at the Tx and Rx to achieve spatial diversity and multiplexing gains. The concert of the overall system, including coverage, capacity, and link quality, is influenced by the gain response of the antennas. A higher gain antenna enables more robust signal transmission and reception, improving signal-to-noise ratio (SNR), increasing system capacity, and extending coverage range. Moreover, the gain response affects important MIMO channel characteristics such as channel correlation and spatial efficiency, directly impacting data rate and system reliability. Therefore, optimizing the gain response is grave for maximizing the concert of MIMO systems^41^.

Enhancing gain in a MIMO antenna system involves a comprehensive approach to maximize signal reception and transmission. The first and most important aspect is selecting the proper shape of the patch and ground shape to help achieve high gain with broader radiation patterns that can focus the signal energy in specific directions. Proper placement of these antennas is crucial. Elevate antennas to reduce interference among radiating elements. Maintain an optimal separation distance between MIMO antennas, which varies depending on the frequency and antenna characteristics. Align antennas according to their polarization, radiation patterns, and proper insertion of parasitic elements among radiating structures. The careful antenna selection and placement are foundational steps to improving gain^42^. Another aspect is choice of frequency bands can impact antenna gain. Higher-frequency bands, such as millimeter-wave frequencies, often enable the use of smaller, higher-gain antennas. Carefully select the frequency band that aligns with your application's requirements and regulatory considerations. Enhancing gain in a MIMO antenna system requires a holistic approach that considers antenna selection, placement, spatial diversity, beamforming, and frequency band selection. By carefully addressing each of these aspects, you can significantly improve the gain of your MIMO system, resulting in better signal reception and transmission capabilities in wireless communication scenarios^43^.

**Figure 4:**
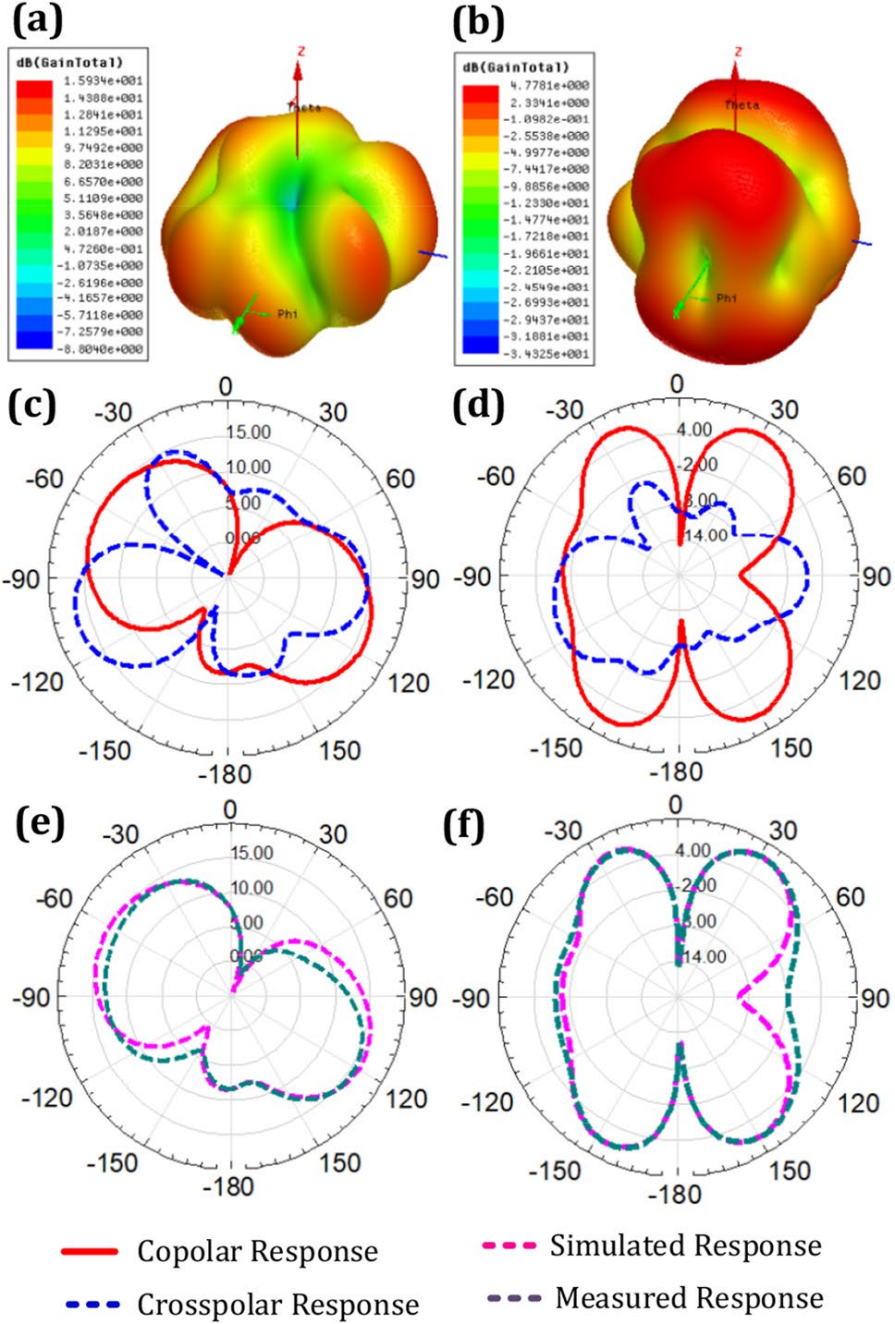
(**a**) 3D gain response of four port MIMO antenna at 3 GHz. (**b**) 3D gain response of four port MIMO antenna at 5.43 GHz. (**c**) Co and cross plot response four port MIMO antenna at 3 GHz. (**d**) Co and cross plot response four port MIMO antenna at 5.43 GHz. (**e**) Radiation Patten response of four port MIMO antenna at 3 GHz. (**f**) Radiation Patten response of four port MIMO antenna at 5.43 GHz.

The presentation of the planned four-port MIMO antenna in terms of gain and radiation pattern is represented in Fig. 4. Figure 4a represents 15.93 dB of gain response for 3 GHz. The gain response of 4.77 dB was achieved for the resonance of 5.43 GHz, as shown in Fig. 4b. The terms co-polar and cross-polar in MIMO relate to the polarization states of the antennas. When two antennas are horizontally or vertically polarized, they are said to be co-polar. However, cross-polar antennas have their polarizations oriented in opposite directions, with one antenna being horizontally polarized and the other vertically polarized. Because of their effect on MIMO system performance, co-polar and cross-polar have meaning and relevance. A system’s enhanced capacity and performance may be attributed to the co-polar antenna's superior signal coupling and channel correlation. However, cross-polar antennas provide more spatial variety, which lessens the impact of fading and boosts system dependability. By striking a good balance between diversity gains, capacity, and link quality, the MIMO system's total performance may be optimized by carefully selecting and placing co-polar and cross-polar antennas^44,45^. The co-polar and cross-polar response of the planned four-port MIMO antenna for the 3 GHz resonating frequency is observed in Fig. 4c, and for the 5.43 GHz resonance is shown in Fig. 4d. The judgement of simulated and measured radiation response for the resonance of 3 GHz is observed in Fig. 4e, and the resonance of 5.43 GHz is observed in 5.43 GHz, as shown in Fig. 4f. Overall the healthy similarity among both results is analyzed based on the response.

**Figure 5:**
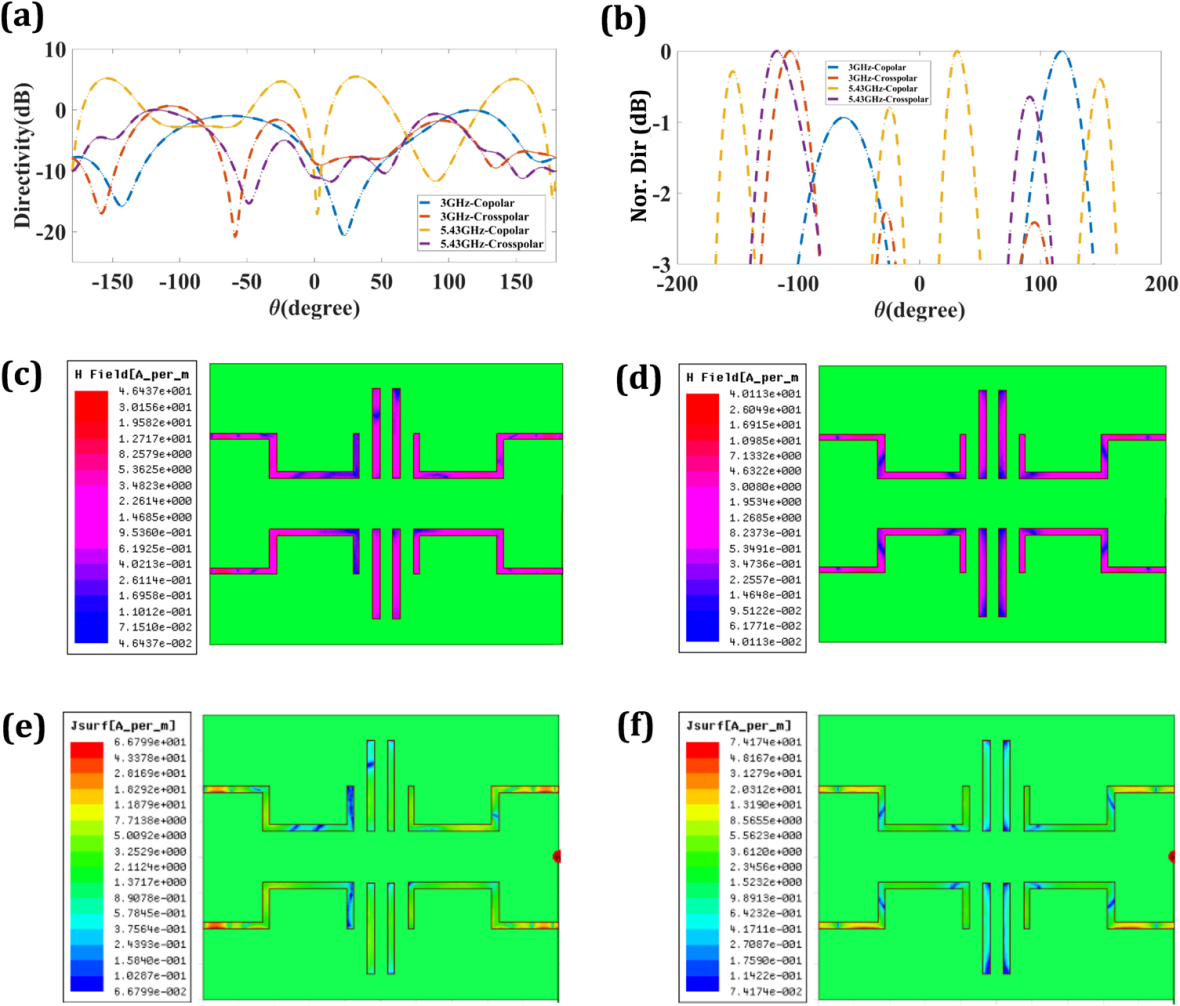
(**a**) Copolar and cross-polar directivity response of proposed four port MIMO design. (**b**) Normalized directivity response of proposed four port MIMO design. (**c**) E-Field for the resonance of 3 GHz. (**d**) E-field for the resonance of 5.43 GHz. (**e**) Jsurf for the resonance of 3 GHz. (**f**) Jsurf for the resonance of 5.43 GHz.

In wireless communication systems, the directivity response of an antenna is crucial. It describes an antenna’s directional bias, indicating how well it can send or receive signals in that direction. Increases in signal intensity and communication range in the desired direction are achieved by increasing directivity, which causes the radiation pattern to become more concentrated. Long-distance communication, expansive signal coverage, and high-quality links all need to be directed energy. It improves the system's overall performance by allowing for more precise regulation of signal propagation and lessening the impact of interference. Directivity enhances the signal-to-noise ratio (SNR) and the overall signal quality of the system by focusing most of the energy in the desired direction. As a result, maximizing an antenna's directivity response is essential for reliable and effective wireless communication^46,47^. The co-polar and cross-polar directivity response of the planned design is observed in Fig. 5a. The half-power directivity for 3 GHz and 5.43 GHz resonating frequencies is shown in Fig. 5b. The 3 GHz of resonance represents the peak co-polar value observed at 117°, and the cross-polar value is observed at − 108°. The 5.43 GHz of resonance represents the peak co-polar value observed at 31°, and the cross-polar value at − 118°. The half-power directivity response is observed in Fig. 5b. The half-power co-polar response for 3 GHz resonance frequency was observed for 29° (− 159 to − 130), and 40° (4 to 44). The cross-polar response for the 3 GHz of resonance frequency observed for 25° (− 173 to − 148), 16° (− 67 to − 51), and 5° (133 to 137). The half power co-polar response for 5.43 GHz resonance frequency observed for 11° (173 to − 178), 11° (− 3 to 7), and 41° (70 to 111). The cross-polar response for the 5.43 GHz of resonance frequency observed for 5° (− 180 to 177), 20° (− 59 to − 39), 25° (− 5 to 20), 6° (49 to 55), and 14° (149 to 163).

Knowing and optimizing MIMO systems requires knowing MIMO antenna E-field distribution. MIMO technology transmits and receives signals on distinct spatial streams using multiple antennas at both ends. The E-field distribution shapes the spatial features of these transmitted signals, affecting system performance. MIMO antenna E-field dispersion matters for numerous reasons. First, it impacts MIMO diversity gains. Spatial variety reduces fading and improves signal degradation by regulating the E-field distribution. The antennas' E-field dispersion enhances signal separation and reliability. Second, spatial multiplexing advantages depend on E-field distribution. Spatial multiplexing sends numerous data streams over the same frequency range using separate antenna components. The E-field distribution separates and couples various streams, increasing data speeds and system capacity. Optimizing E-field distribution improves signal coverage and reduces interference. The E-field distribution may be modified to concentrate energy on desired coverage regions by adjusting antenna directionality and radiation pattern, boosting signal strength and quality. E-field distribution management may also reduce interference from nearby antennas or signal sources, guaranteeing a stable communication connection. FEM or MoM numerical electromagnetic simulations are used to analyze and optimize MIMO antenna E-field distributions. These simulations reveal MIMO system spatial properties and performance, enabling effective antenna design and layout. In conclusion, MIMO antenna E-field distribution affects diversity gains, spatial multiplexing gains, coverage, interference reduction, and system performance. MIMO systems may enhance wireless communication data speeds, capacity, and reliability by understanding and optimizing E-field distribution^48^.

Checking isolation based on surface current density involves calculating the current flowing along the surface of conductive materials, dividing it by the cross-sectional area, and comparing the resulting surface current density against acceptable thresholds defined by specifications and regulations. This evaluation aims to prevent interference and ensure proper isolation between components^49^. In the proposed design passive elements located between the two radiating patches enable the isolation in the structure. The proposed design is resonating at 3 GHz and 5.43 GHz. It is very clear from the graph that the surface charge distribution is not able to pass from one radiating element to another radiating element due to the passive structure. Figure 5c represents 46.3 A/m field distribution at 3 GHz resonating frequency. Figure 5d represents 40.11 A/m field distribution at 5.43 GHz of resonance. The field distribution varies according to the variation of the resonance frequency. The Jsurf for the resonance of 3 GHz is 66.7 A/m and 5.43 GHz is 74.1 A/m as shown in Fig. 5e and f.

**Figure 6:**
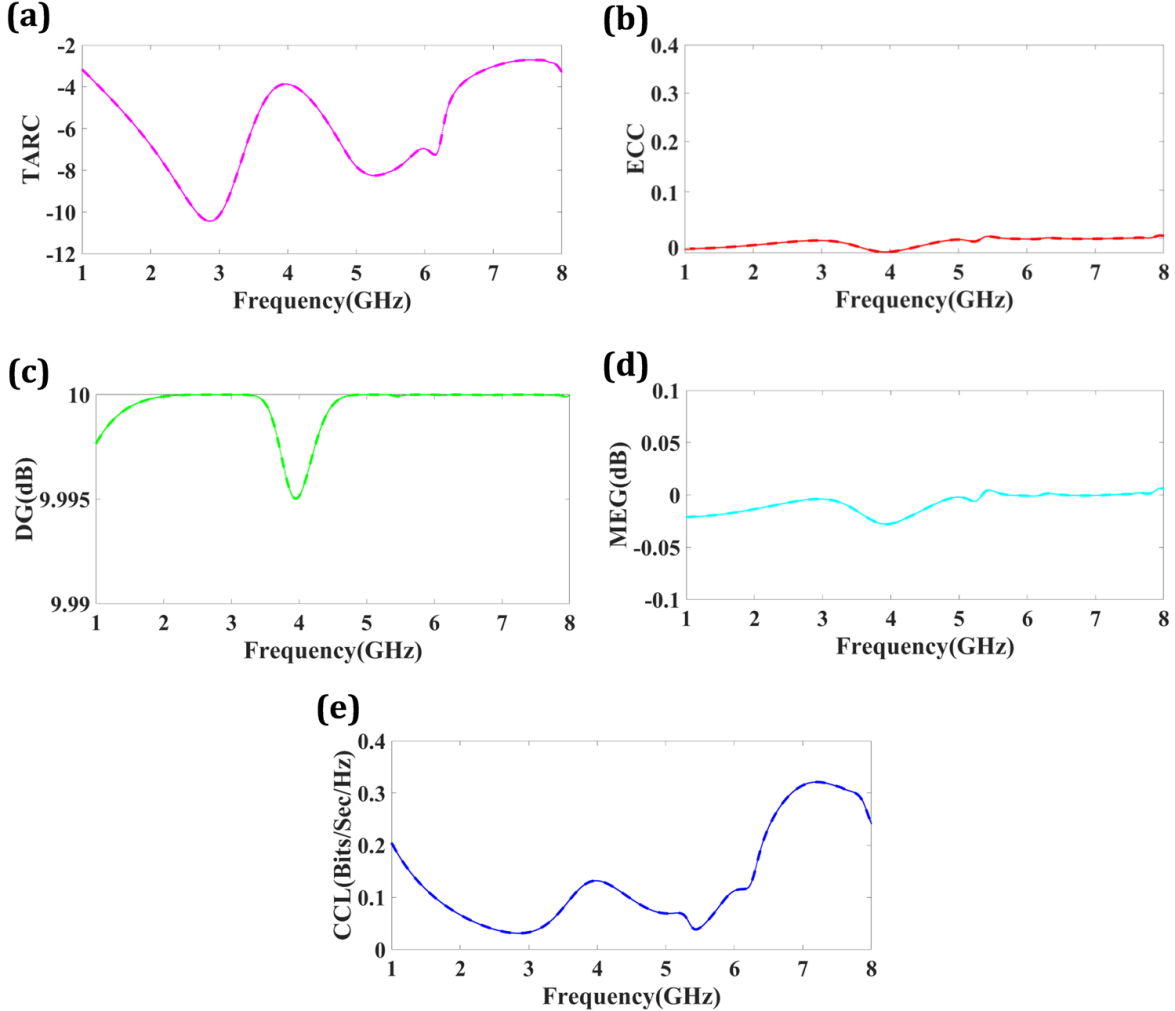
(**a**) TARC result of proposed four port structure. (**b**) ECC result of proposed four port structure. (**c**) DG result of proposed four port structure. (**d**) MEG result of proposed four port structure. (**e**) CCL result of proposed four port structure.

MIMO antenna systems' impedance matching and radiation efficiency are determined by the TARC (Total Active Reflection Coefficient). The TARC optimizes MIMO system performance by transmitting and receiving signals concurrently on separate spatial streams. TARC combines the antenna’s reflection coefficient and radiation efficiency. A low TARC implies effective antenna impedance matching, minimizing reflected power and maximizing power flow to the transmission medium. This optimizes power transmission and decreases system losses. MIMO systems need low TARC to preserve signal quality and reduce antenna interference. Common TARC antennas are well-matched to the transmission line and feature low signal reflections. This boosts system capacity and data throughput by lowering antenna interference. Low TARC also improves antenna radiation efficiency. Antenna radiation efficiency is how well it converts electrical energy into electromagnetic waves. Low TARCs improve antenna radiation efficiency by minimizing reflections and maximizing power transmission, producing more robust and dependable signals. MIMO antenna design, impedance matching, and matching network selection optimize TARC. By reducing the TARC, antennas may increase signal transmission and reception, interference, capacity, coverage, and dependability. Finally, the TARC in MIMO antennas affects impedance matching, power transfer efficiency, radiation efficiency, and interference reduction. MIMO systems may improve wireless data speeds and performance by optimizing the TARC. Equation (6) can be used to calculate the TARC. The negative value of TARC is observed in Fig. 6a. The TARC result is within the allowed range.

6 $$ TARC = \frac{{\sqrt {\left[ {\left| {\left( {S_{11} + S_{12} e^{j\theta } } \right)} \right|^{2} } \right] + \left[ {\left| {\left( {S_{21} + S_{22} e^{j\theta } } \right)} \right|^{2} } \right]} }}{\sqrt 2 } $$

The Envelope Correlation Coefficient (ECC) may assess signal envelope correlation between antenna components. Signal envelope magnitude is independent of phase. The Equal Channel Correlation (ECC) statistic measures signal envelope similarity across antennas. When the ECC is approaching 1, several antennas provide less spatial variety. It shows closely linked antenna signals. However, a small ECC indicates that the antennas’ signals are independent, maximizing spatial diversity gain. The ECC may be calculated using power levels and antenna component correlation coefficients. Cross-correlation and auto-correlation techniques may be used to calculate the correlation coefficient, a linear measure of antenna signals. Reflections and dispersion from nearby objects may raise ECC, antenna, and mutual coupling. The MIMO system may lose spatial diversity if these factors induce unwanted correlations between antenna signals. The ECC may fine-tune a MIMO system's design and execution to increase wireless communication dependability and quality. ECC response of less than 0.5 improves communication. Equation (7) calculates the ECC response. Figure 6b represents ECC response is near zero. The lower values indicate less correlation between antenna components, which means MIMO’s superior performance.

7 $$ ECC = \frac{{\left| {S_{11}^{\rm { \star }} S_{12} + S_{21}^{\rm { \star }} S_{22} } \right|^{2} }}{{\left( {1 - \left( {\left| {S_{11} } \right|^{2} - \left| {S_{21} } \right|^{2} } \right)} \right)\left( {1 - \left( {\left| {S_{22} } \right|^{2} + \left| {S_{12} } \right|^{2} } \right)} \right)}} $$

where Complex conjugate is denoted by *, the Far filed method for calculating ECC is not proper; therefore S-Parameter is a better choice for the analysis^50^. MIMO systems can outperform single-antenna systems by leveraging the wireless channel’s spatial dimension. MIMO systems benefit from diversity gain (DG) by employing many antennas at the Tx and Rx to increase signal quality. MIMO sends and receives signals over a standard frequency band using multiple antennas. Wireless signals may fade and attenuate due to many propagation channels. Multiple antennas reduce fading and improve system performance. Calculating DG involves antenna number, correlation, and channel conditions. Several antennas and channel variations increase diversity gain. Uncorrelated channels maximize diversity gain, whereas heavily correlated channels minimize it. Beamforming, precoding, and space–time coding may increase the diversity advantage of MIMO. MIMO diversity gain may improve reliability, capacity, and coverage in wireless communication systems with several antennas.

8 $$ {\text{DG}} = 10\sqrt {1 - ({\text{ECC}})^{2} } $$

The DG calculation is done using Eq. (8). As shown by the Equation, there is a negative correlation between DG and ECC. DG values for all of the designs were almost 10 dB as shown in Fig. 6c. MIMO antenna systems depend on Mean Effective Gain (MEG). It characterizes an antenna’s average gain in several directions by considering its radiation pattern and the MIMO system’s spatial correlation. The MEG directly affects MIMO system capacity and network performance. The antenna's emission pattern and MIMO channel spatial properties determine its effective gain. MIMO system capacity and performance depend on antenna correlation. Low correlation improves spatial multiplexing, whereas high correlation reduces spatial variety and ability. The MEG considers spatial correlation, improving performance evaluation. Optimizing the MEG improves MIMO system performance. Higher MEG antennas improve signal transmission and reception, increasing signal quality, SNR, and system capacity. It optimizes data rate and geographical resource use. MEG is calculated using the antenna radiation pattern, spatial correlation between antennas, and MIMO channel statistical features. Channel modelling, antenna design, and optimization algorithms optimize the MEG and MIMO systems. MIMO antennas' Mean Effective Gain (MEG) controls radiation pattern and spatial correlation, affecting system capacity and connection performance. Figure 6d shows the proposed structure's MEG response. The MEG response is near zero over the entire spectrum.

The importance of MIMO channel capacity loss in determining the bounds and influencing variables of the maximum capacity of MIMO systems is critical. CCL can be calculated using Eqs. (9)–(11). MIMO channels experience capacity loss for several reasons, including but not limited to channel correlation, fading, noise, interference, and incomplete knowledge of the channel state. For MIMO systems to function at their full potential, it is crucial to identify and solve these issues. MIMO communication systems’ overall efficiency and data rate may be enhanced by using methods like antenna selection, transmit beamforming, and sophisticated signal processing algorithms to reduce capacity loss. This results in more efficient and dependable wireless communication.

9 $$ C_{loss } = - log_{2} det\left( {\zeta^{v} } \right) $$

10 $$ \zeta^{V} = \left[ {\zeta_{11} \;\zeta_{12} \;\zeta_{21} \;\zeta_{22} } \right] $$

11 $$ \begin{aligned} \zeta_{11} & = 1 - \left[ {\left| {S_{11} } \right|^{2} + \left| {S_{12} } \right|^{2} } \right], \,\zeta_{22} = 1 - \left[ {\left| {S_{22} } \right|^{2} + \left| {S_{21} } \right|^{2} } \right], \\ \zeta_{12} & = - \left[ {S_{11}^{*} S_{12} + S_{21}^{*} S_{12} } \right], \;\zeta_{21} = - \left[ {S_{22}^{*} S_{21} + S_{12}^{*} S_{21} } \right]. \\ \end{aligned} $$

The CCL response is less than 0.3 for the proposed frequency span, as shown in Fig. 6e. The CCL response is within the allowed limit. The performance observation for one-port design, optimized one-port, two-port and four-port designs are presented in Table 1 for comparing return loss, bandwidth and resonating frequency.

**Table 1:** The performance observation of proposed one-port, optimized one-port, two-port and four-port MIMO antenna designs.

Number of ports	Return loss (dB)	Resonating frequency (GHz)	Bandwidth (GHz)
1	− 18.77	3.825	0.73 (3.445–4.175)
1-Optimized	− 18.65	3.935	0.82 (3.42–4.24)
2	− 19.79	3.835	0.71(3.515–4.225)
4	− 20.53	3	1.375 (2.14–3.515)
	− 12.37	5.43	0.25 (5.335–5.585)

The design performance of the presented structure with other articles is shown in Table 2. The proposed design provides a better gain with healthy diversity performance than different designs.

**Table 2:** The performance comparison among different published articles with the presented design.

References	Operating frequency band (GHz)	Gain (dB)	ECC	DG (dB)
Presented Work	2.14–3.515 5.335–5.585	15.93	~ 0.01	~ 9.99
^51^	2.2–3.55, 2–5.8	4	< 0.05	–
^52^	3.1–10.6	6.5	< 0.5	~ 9.95
^53^	3.1–10.6	6.5	0.2	–
^54^	2.38–2.52, 3.28–3.62, 5.05–6.77	1.5–4.5	0.005	~ 9.75
^55^	2.3—2.9	5.1	< 0.15	~ 9.6
^56^	3.3–7.88	4	< 0.05	~ 9.9
^57^	2.2–6.28	4	< 0.25	–
^58^	2.70–4.94	4	< 0.1	–
^59^	1.8–2.9	6.4	< 0.10	9.97
^60^	30/38	8.4	0.001	9.8
^61^	2.5–30	8.75	–	~ 9.7
^62^	3–12	6.2	< 0.001	~ 9.9
^63^	4–17	5.87	< 0.02	–
^64^	7–20	7.68	–	–
^30^	3.26–5.18	3.51	< − 0.005	∼10.0
^65^	3.89–11.97	–	< − 0.4	> 9.95
^38^	3.5–5.6	5.42	0.1	9.9

## Conclusion

The sickle-shaped four-port MIMO antenna structure is presented in the manuscript. The design optimization is achieved by initially designing and optimizing the single port element structure, then preparing for two port elements, and finally, the four-port design is finalized. Three parametric analyses are considered, like variation in length, the width of the sickle-shaped patch, and varying length of DGS. The 2–8 GHz frequency span is taken to analyze the structure. The proposed design is fabricated using low-profile material. The judgment among measured and simulated results is incorporated in the article. The presented four-port design provides a total gain of 15.93 dB, a peak co-polar value of 5.46 dB, a minimum return loss of − 20.53 dB, maximum bandwidth of 1.375 GHz. All diversity parameters like TARC, ECC, MEG, CCL and DG are within the permitted range. She presented a design suitable for the 5G and aeronautical mobile communication applications.

## Data Availability

The data supporting the findings in this work are available from the corresponding author with a reasonable request.
